# Nutrition transition in South Asia: the emergence of non-communicable chronic diseases

**DOI:** 10.12688/f1000research.5732.2

**Published:** 2015-11-24

**Authors:** Ghose Bishwajit

**Affiliations:** 1 Institute of Nutrition and Food Science, University of Dhaka, Dhaka, Bangladesh; 2 Current Address: School of Social Medicine, Tongji Medical College, Huazhong University of Science and Technology, Wuhan, Hubei, China

**Keywords:** South Asia; Nutrition Transition; Epidemiological Transition, Non-communicable diseases; Healthcare

## Abstract

**Overview:** South Asian countries have experienced a remarkable economic growth during last two decades along with subsequent transformation in social, economic and food systems. Rising disposable income levels continue to drive the nutrition transition characterized by a shift from a traditional high-carbohydrate, low-fat diets towards diets with a lower carbohydrate and higher proportion of saturated fat, sugar and salt. Steered by various transitions in demographic, economic and nutritional terms, South Asian population are experiencing a rapidly changing disease profile. While the healthcare systems have long been striving to disentangle from the vicious cycle of poverty and undernutrition, South Asian countries are now confronted with an emerging epidemic of obesity and a constellation of other non-communicable diseases (NCDs). This dual burden is bringing about a serious health and economic conundrum and is generating enormous pressure on the already overstretched healthcare system of South Asian countries.

**Objectives**: The Nutrition transition has been a very popular topic in the field of human nutrition during last few decades and many countries and broad geographic regions have been studied. However there is no review on this topic in the context of South Asia  as yet. The main purpose of this review is to highlight the factors accounting for the onset of nutrition transition and its subsequent impact on epidemiological transition in five major South Asian countries including Bangladesh, India, Nepal, Pakistan and Sri Lanka. Special emphasis was given on India and Bangladesh as they together account for 94% of the regional population and about half world’s malnourished population.

**Methods**: This study is literature based. Main data sources were published research articles obtained through an electronic medical databases search.

## Introduction

**Figure 1.**
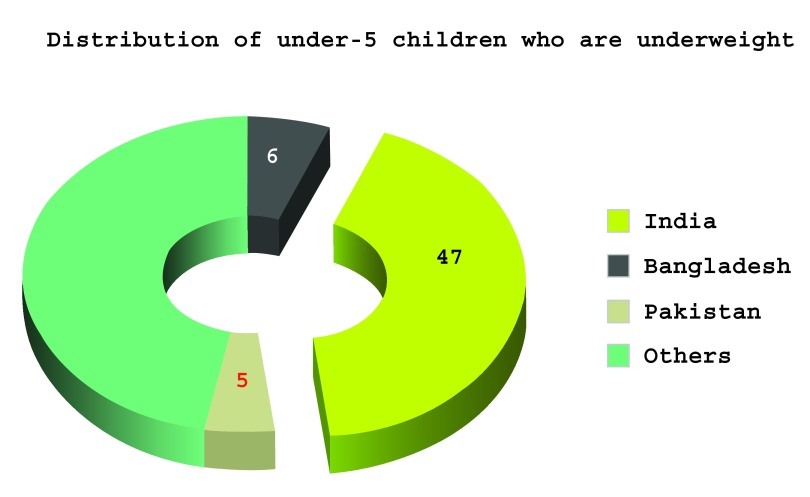
Shows the percent distribution of underweight children in South Asia and the rest of the world. **Source:** Global disease burden 2010 ( http://goo.gl/Tp7fS1)

Globalization of agrifood has brought about remarkable shifts in diet patterns especially in developing countries. Changes in dietary pattern have shown to be a major underlying factor for increasing prevalence of obesity and associated NCDs. With long standing history of infectious diseases, developing countries are now facing a rising tide of non-communicable diseases which is popularly known as the double burden of malnutrition (Coexistence of over- and undernutrition). This dietary transition is basically characterized by a shift from a diet with a higher proportion of carbohydrate based foods such as cereal grains (rice, wheat, maize), vegetables (leaves, roots, legumes) and low animal products (meat, egg, milk) to one which is lower in carbohydrate and higher in animal-based food with high sugar and caloric content and larger amount of processed food (chocolates, soft drinks). Though dietary and epidemiological transition have been two main foci in the study of nutrition transition, more socio-economic and demographic parameters are now being included in the discipline. The term *nutrition transition* was first coined by Barry M. Popkin who remains one of the most highly cited researchers in the field of human nutrition ( Wahdan, 1996). According to Popkin, the scope of nutrition transition encompasses not merely the dietary and physical aspects, but also many other economic, social and environmental factors that is shaping the landscape of modern living as never before. By virtue of increased participation in global trade, South Asian nations have experienced a period of unprecedented economic growth, higher income level, provision of labor-saving technologies, and a significant reduction in the number of people living in extreme poverty. India, the largest economy in the region, rose to global prominence with its trillion dollar economy and is set to be world’s third largest economy by 2050 (World Bank). Despite the global recession, India’s share in global trade increased to 1.28 per cent in 2011 compared to 0.67 per cent a decade earlier (Mubarak, 2012). Bangladesh has earned the recognition of being the economic miracle of the decade and is likely to surpass Pakistan to become the second largest economy in the region. Economic transition with improvement in household economic status has shown to be associated with increased consumption of animal products and higher prevalence of overweight, obesity and other NCDs ( Salter, 2013), and South Asia appears to be no exception in this regard. Since 1990, South Asian countries are experiencing an increasing trend in the prevalence of overweight and obesity ( Popkin *et al.*, 2012). India with around 35% total population living on vegetarian diet ( Michalak *et al.*, 2012) has experienced a doubling in total poultry meat consumption since 2000 while in Pakistan total meat consumption has increased by 130% during the same period ( Tirmizi, 2012). Increased cross-border food trade, advancement in local food technology and in food marketing and processing industry have greatly increased the availability of processed food products even in the remote rural areas where around 70% of the South Asians live. Though chronic dietary (caloric) inadequacy, macronutrient deficiency and infectious diseases are traditionally conceived as the major causes of malnutrition in South Asian countries, the impact of adequate but unhealthy diet along with lifestyle and environmental factors are fast replacing the trend and are becoming the major focus of epidemiologists. Rapid urbanization, access to labor-saving technologies and rise in various non-farm sectors have reduced the need and scope for physical activities to a level a level which is contributing to a sharp rise in the prevalence of overweight and obesity. However, the benefit of economic growth didn’t translate to improved nutritional status for the population at large, a phenomenon which is known as the South Asian enigma (Guha-Khasnobis *et al.*, 2010). More than half of world’s total underweight children live in South Asia [ Figure 1]. While malnutrition is the single largest cause of child mortality in the region, childhood obesity is also becoming a public health concern especially in the urban areas ( Roux *et al.*, 2008). Thus at one the end of the epidemiological spectrum lies widespread undernutrition, and a rising tide of obesity and associated NCDs on the other. This rising dual burden of disease is posing enormous pressure on the underdeveloped healthcare systems in South Asia and provides an imperative to adopt more mainstreaming and cross-cutting policies in national and regional level.

## Methodology

This study is based on literature published between 1995 and 2014. A systematic literature review was conducted in April of 2014 in the following electronic databases: PubMed, Embase, The Cochrane Library and Google Scholar; using the following search terms: ‘nutrition transition’, ‘dietary transition’, ‘epidemiological transition’, ‘obesity’, ‘diabetes’, cardiovascular diseases. Main inclusion criteria was relevance to studies in context of following South Asian countries: India, Pakistan, Bangladesh, Nepal and Sri Lanka. Following the preliminary search, the titles and abstracts of the articles were analysed and selected according to their suitability for this review. Both original and review articles were included. Original studies including larger sample sizes and sound methodology, and review articles based on broad set of studies (case-controlled, cross sectional, randomized trials) were given priority in the selection of studies. Owing to scarcity of relevant studies no special exclusion criteria was applied except for availability of full text articles. Additional data were triangulated from various sources mainly from FAO, USDA, WHO, World Bank, and health and nutrition surveys in each countries.

## Demographic transition in South Asia

**Figure 2.**
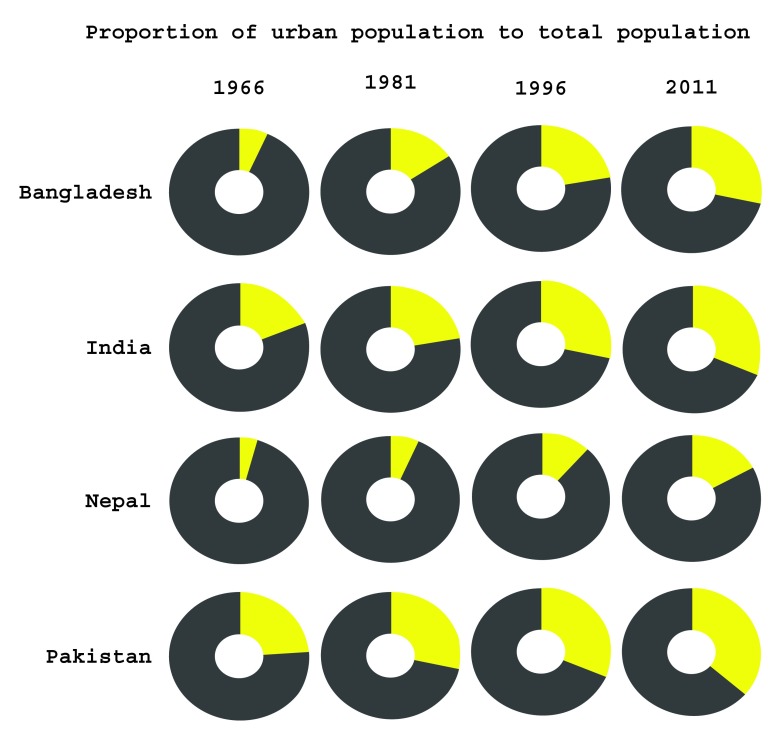
Illustrates the trend of urbanization in South Asia. Pakistan has highest urban population in the region while Nepal has the highest rate of urbanization. **Source:** UN Population Database, State of world cities ( http://goo.gl/6G7S3h)

South Asian demography is characterized by declining fertility and mortality rate [ Table 1], high population density (seven times the world average), and a large youth segment. It houses around one fifth of global population with less than 4% of global land area and contributing to merely 2% of global income ( Jacques, 2008). Total population was 1.65 billion in 2012 of which 31% are located in urban settings. Though most people are living in the rural areas, urbanization is occurring at a great speed [ Figure 2]. Three of world’s top ten most populous countries (India, Pakistan, Bangladesh) and four of top ten most populous cities are located in this region (Mumbai, Karachi, Delhi, Dhaka). South Asia has registered remarkable progress towards the Millennium Development Goals (MDGs) [ Table 1] and has substantially reduced the rate of premature death and disability from infectious diseases such as polio, pneumonia, diarrhea and many malnutrition related deaths thanks strong government commitment and a steady economic growth. In Bangladesh for example, total infant and child mortality rate has decline respectively by around 60 and 50 per cent since 1980. All countries are experiencing increasing trend life expectancy. India accounts for around 75% of total population in South Asia and contributes to around 80% of regional GDP. India’s population surpassed billion mark in 2001 and is likely to exceed China to become the most populated country by 2050 ( Doak *et al.*, 2005; World Bank, 2013). Bangladesh, the most densely populated nation in the world, is the third largest country in South Asia and eighth in the world in terms of total population. Population growth rate declined from 2.1% to 1.4% since 1990s which provides a concrete example of how economic growth is linked with decline in population growth, increased life expectancy and reduced child mortality ( Bleich *et al.*, 2011; Basu *et al.*, 2013). The country has made good progress in all areas of human development after a long history of huger, food insecurity and human deprivation. Following decades of internal conflicts, Nepal and Sri Lanka have been able to restore peace and have also achieved great success in health and economic terms. Population growth rate is about 0.7% in Sri Lanka and is the least populated country in the region with a population of 20.33 million in 2012. By virtue of this sharp fall in infant mortality rates, the size of the child and youth segment is also expanding. South Asia already has about a quarter of world’s youth and is also the most youthful sub-region in Asia (32% population are below 14). Though its a good sign for the economy as more people are being able to join the workforce to supply cost-effective labor for the fledgling economy, the demographic transition is actually serving as a double-edged sword as it has also produced a rapid increase in numbers of older people who are more prone to NCDs and largely account for the rapidly increasing NCD burden ( Basnyat & Rajapaksa, 2004). Besides that, this huge youth population is also being exposed to the growing obesogenic environment and a multitude of other risk factors of NCDs while striving to create the conditions for sustainable careers and quality living for themselves. Childhood obesity has shown to be associated with an increased risk of NCDs in later years ( Ghose *et al.*, 2013). Since aging population bears increased susceptibility to NCDs, the total burden from these diseases may reach a huge proportion in next two decades, if steps are not taken immediately to avert this situation.

**Table 1.** Trend in selected economic and development indicators in South Asia.

**Year**	**1960**	**1970**	**1980**	**1990**	**2001**	**2011**
**IMR**	146	130	115	89	70	48.30
**Life Expectancy**	46	48	60	58	62	NA
**GDP**	83.39	120.54	264.10	358.98	448.01	1,386.06
**Prevalence of Underweight in preschool** **children (%)**	58.1	54.5	50.9	47.3	43.6	NA
**Mortality rate, under-5 (per 1,000 live births)**	241.35	194.50	153.90	118.60	86.20	62.10
**Population density**	121	150	189.74	239.94	292.97	347.18
**Primary enrollment rate (%) of primary** **school age children**	NA	57.39	63.84	73.09	78.04	92.50

**Source: FAO, HDI 2013**

Table 1 shows the demographic and economic trends in South Asia. It is evident from this table that income has increased dramatically with a sharp rise in 2011. Infant mortality and net under-five mortality have also declined substantially over past three decades with a significant rise in life expectancy. This demonstrates how economic progress is linked with the improvement in health status of countries. (NA- Data not available)

## Drivers of nutrition and dietary transition and their impact on NCDs

A dietary transition from high starch and low animal food to higher intake of animal based food, higher proportions of edible oils and sweetener are occurring in almost all developing regions. Nutrition transition is occurring as an inevitable outcome of the changes in global food system triggered mainly by increasing income levels, rapid industrialization and urbanization and globalization of agrifood.

### Income

Historically, nutrition and dietary transition is found to be associated with a shift from a preindustrial agrarian economy to an industrial economy as seen in the context of developed countries in Europe and North America ( Barry, 1999). Industrialized countries have experienced a nutritional and epidemiological transition since the industrial revolution. Reform in economic system brings about changes in food system, and when accompanied by modification in lifestyle habits such as reduced degree physical activity, it contributes to changing disease pattern such as rising prevalence of overweight, obesity and associated NCDs ( Doak *et al.*, 2005).

**Figure 3.**
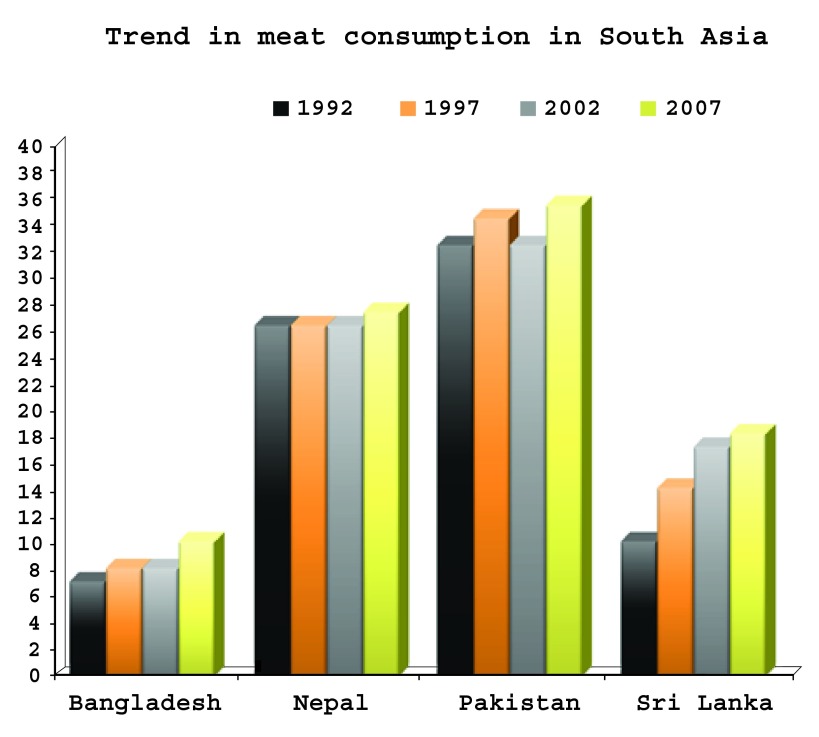
Shows the trend in total meat consumption trend in South Asia over the past two decades. Per capita meat consumption rate (shown on the Y-axis) has increased in almost all countries with Sri Lanka having the highest rate. Pakistan has the highest per capita meat consumption and is currently world’s tenth largest consumer of beef. **Source:** USDA ( http://goo.gl/p0dehO), Thepoultrysite ( http://goo.gl/Slh83n)

Today, people in developing countries can afford more calories than ever before which is largely attributable to increased disposable income and greater availability of food (Josef *et al.*, 2005). As the demand for food is rising in the aggregate, a major shift is also taking place in the type of food demanded. This shift generally occurs to increased demand for fish, egg, meat and dairy based food, and poor households tend to shift to more animal based diet with the rise in income ( Misra & Shrivastava, 2013; Singh *et al.*, 2011). Figure 3 shows that total meat consumption has increased significantly in all South Asian countries over past two decades. Though over 60% of all South Asians still earn less then $1.25 a day, the situation is actually much better compared to three decades back and more and more people are being able to afford these type of food. Per capita GDP growth has seen a dramatic leap since 2001 [ Table 2] which has played a catalytic role in increasing the demand for meat, fish, egg, and dairy products (Freedman, 2002). Many researchers attributed the food price inflation of 2007–08 to the rising demand from India and China as the countries are becoming major food importers to meet huge domestic demand ( Ghose, 2014).

**Table 2.** Sectoral share of employment in selected South Asian countries (% of total employment).

	Agriculture		Service	
	1985	2005	1985	2005
**Bangladesh**	57.70	48.10	26.00	37.40
**Pakistan**	50.60	43.00	28.70	36.60
**Sri Lanka**	49.30	30.70	27.90	38.40

**Source: CIA Factbook ( http://goo.gl/SqSOTB)**

Table 2 illustrates the shift in percent labour force allocation in selected South Asian countries. Despite being agrarian economies, total employment in agriculture has declined in all countries, while that in service sector has increased.

### Urbanization

Though around 60% of people are living in the rural areas, all South Asian countries are experiencing a very fast urbanization [ Figure 2]. Prevalence of obesity is higher in urban areas as compared to the rural areas, since these are most affected by rapid changes in nutritional pattern and sedentary life style ( Shariful Islam *et al.*, 2013). Rapid urbanization has been associated with a shift in labor force from agriculture to service sector which has reduced average physical activity status of the population. Table 3 shows that people are becoming more involved in service jobs in South Asian countries. For example in Bangladesh, prevalence of diabetes in urban areas is twice as high as in the rural areas ( Saquib *et al.*, 2012). Dhaka is one of the fastest growing cities in the world which is due mainly to the rapid growth of the garments sector attracting cheap labor from rural areas. Nepal is leading the race of urbanization in South Asia with a rate of 4.9% while that for Sri Lanka is the lowest (0.7%). From a dietary point of view, urbanization is characterized by a marked increase in the intake of energy-dense foods, a decrease in physical activity, and a heightened level of psychosocial stress, all of which promote the risks of developing metabolic syndrome ( Barry, 1999). Urbanization affects people’s health and diet in various ways. People living in urban areas consume diets distinctly different from those of their rural counterparts which usually includes an increasing amount of animal and dairy products, sugar rich food, fast foods and less of fruits and vegetables ( Barry, 1999). People living in urban areas not only show an increased appetite for processed and convenience foods and diets high in sugar and fat content, but also a more sedentary lifestyle which sets the preconditions for obesity and most other NCDs ( Barry, 1999). Urbanization is also accompanying increased female labor force participation. As more and more women are joining the labour force, it is very likely that households will have to rely more on precooked convenience food and fast food rather than home cooked traditional food due to increasing time constraints. A substantial increase in childhood as well as adult obesity in the urban population is therefore in line with the radical changes in lifestyle during the last few decades ( Han *et al.*, 2010).

**Table 3.** Proportional mortality from NCDs in South Asian in 2008 (% of total deaths, all ages).

	CVD	Cancer	Diabetes	Other NCDs	Total
**India**	24	6	2	21	53
**Bangladesh**	27	9	2	14	52
**Pakistan**	25	7	1	13	46
**Nepal**	25	11	2	13	51
**Sri Lanka**	30	9	4	22	65

**Source:** Global Health Observatory Data. WHO

Table 3 shows that NCDs account for highest causes of mortality in South Asia. Among all NCDs, contribution of cardiovascular diseases is the highest followed by cancer and diabetes.

### Globalization and the changing food system

**Figure 4.**
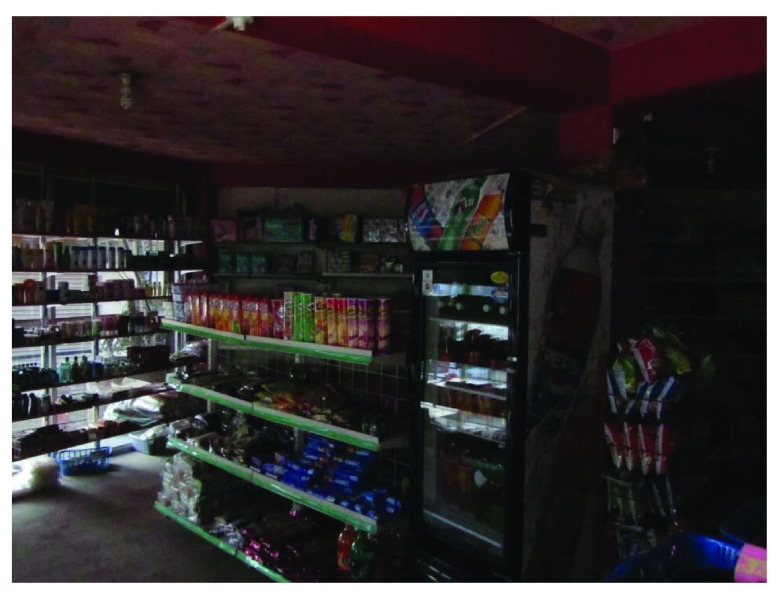
This photo was taken from a super-shop in Janakpurdham district in Nepal. Janakpurdham is known for many historical and religious sites and has very little traces of industrialization. **Photo credit: Mr. Sudeep Sharma**

There is a growing evidence that globalization and trade liberalization have played a key role in the dietary and nutrition transition in the developing countries ( Ghose *et al.*, 2013; Lim, 2012). With the advent of food biotechnology and the expansion of transnational food corporations (TFCs), global food chain has undergone massive changes in terms of the ways food is grown, processed, stored, transported and consumed. Food mile (The distance that food travels from the place of production) has reached continental scale as a result of improved transportation system and declining trade barriers. Following the Uruguay Round, the average tariff on most goods fell from about 40% in 1947 to 4.7% in 1993 (Paul, 2008). As a consequence, developing countries have enjoyed a huge inflow of processed food products which has remarkably transformed the scenario of food market and people’s food choices. Nutritionists and health researchers around the world are expressing serious concerns as food is being traded the same manner as garments, electronics, and all other products ignoring the long term health and environmental impacts. Historically in South Asia, community food markets have been the main sources of everyday food supply which are mostly locally grown fresh raw foods. The situation has been changing as groceries and super-shops are taking the lead which are usually selling cheap junk foods such as cookies, chips and soft drinks. Even two decades earlier popular processed items were limited to rice cakes, sweetmeats and vegetable/fruit jelly/pickle which are produced using traditional methods mostly by rural households who has no knowledge of artificial food chemicals. However, this culture is on the verge of ruin with the progress of industrialization as these foods are now being manufactured on an industrial scale by food companies which are dependent on a range on food additives. Large supermarkets which are characteristics of affluent societies are now spreading fast across developing regions like South Asia ( Reardon *et al.*, 2003). Figure 4 shows the interior of a supermarket in a small town in Nepal teeming with soft drinks, cookies, chips. As a result, children especially in the urban areas are increasingly being exposed a completely new food environment which offers cheap availability of numerous food products which are most often obesogenic and have many potential health hazards.

## Epidemiological transition in South Asia and the burden of NCDs

Epidemiological transition refers to the shift from acute infectious and deficiency type diseases to chronic non-communicable diseases (NCDs) which reflects changes in the pattern of morbidity and mortality [1, ( Doak *et al.*, 2005). In general, this transition occurs through a complex pathway which is influenced by gradual changes in nutritional, demographic and socio-economic parameters ( Doak *et al.*, 2005). In industrialized countries, epidemiological transition emerged towards the early 1900s marked by a rising levels of NCDs and a drastic fall in the prevalence infectious disease (Detels, 1997). This rising prevalence NCDs is believed to be an outcome of a complex interplay between a demographic ( Stuckler, 2008), socio-economic ( Thakur *et al.*, 2011), nutritional, environmental factors along with changes in lifestyle pattern ( Popkin *et al.*, 2012). Once known as the diseases of the wealthy western countries, NCDs are fast becoming more threatening in the developing regions such as Latin America and South Asia. Globally, NCDs have become the leading causes of morbidity and mortality (Accounting for 43% of all disease burden in 1999) and is projected overtake that of infectious diseases within a decade (Wagner *et al.*, 2012).

**Figure 5.**
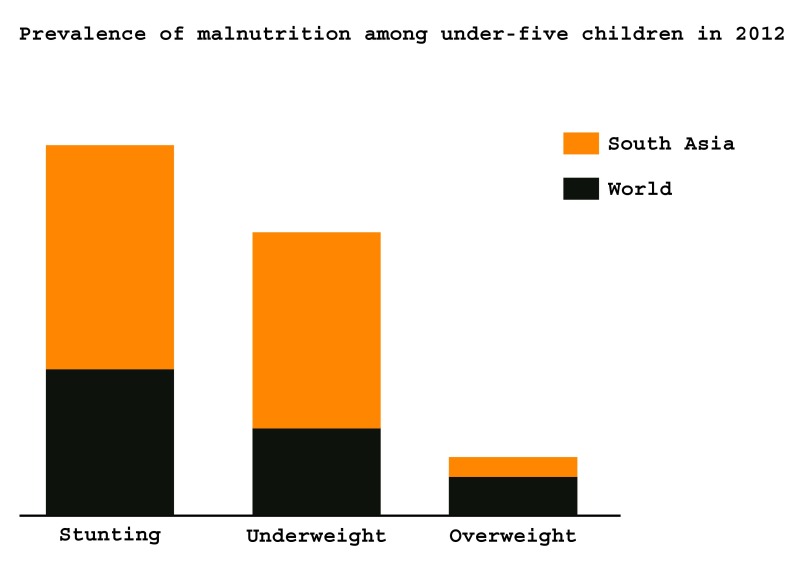
Illustrates the situation of double burden of malnutrition (DBM) in South Asia. The proportion of stunting and underweight is higher than rest of the world, while that of overweight is about one-third. **Source:** WHO, UNICEF ( www.childinfo.org)

In contrast with developed countries where the disease burden is dominated by NCDs (with lower proportion of infectious diseases), countries in the developing world are facing a double burden of malnutrition (Paul, 2008). Like other developing regions, South Asian countries are also facing a double burden of malnutrition ( Haddad *et al.*, 2015) [ Figure 5]. In 2000, 44% of the burden of disease in this region measured in disability adjusted life years (DALYs) was attributed to non-communicable diseases ( Basnyat & Rajapaksa, 2004). According to WHO guidelines for Asian populations, a third of the South Asian adult population is classified as obese, and 50% as overweight, by 2030 NCDs (T2DM, CVDs, COPD, cancer) and are going to account for 72% of total mortality by 2030 in South Asia ( Shariful Islam *et al.*, 2013; Singh *et al.*, 2011). In India, although under-nutrition and micronutrient deficiencies continue to be major public health problems, over-nutrition and obesity are also emerging as a major problem in many states(Kerala, Tamil Nadu). In Bangladesh, NCDs are responsible for almost half of annual mortality and of 41% total disease burden ( Bleich *et al.*, 2011).

South Asians have a higher prevalence of diabetes mellitus than in any other region in the world ( Jafar, 2006) and also have an increased susceptibility to cardiovascular disease (CVD). CVD is the largest single cause of mortality in all South Asian countries [ Table 2]. In Bangladesh, total mortality from NCDs increased by 68% between 1986 and 2006 while that from communicable diseases rose by 5% during the same period ( Ahsan Karar *et al.*, 2009). Bangladesh alone accounts for 40% of all diabetes patients located in least developed countries and the number is increasing by 5–6 percent a year ( Ramachandran *et al.*, 2008). An estimated 7 million people are suffering from diabetes in Bangladesh, while the corresponding figure is respective 2 and 1 million for Nepal and Sri Lanka. In Pakistan, about 12.9 million (10% of total population) people are currently living with diabetes which is projected to become 14.5 million in 2025. The scenario of double burden of malnutrition is perhaps most vivid in India than any other place in the world. India has the recognition of housing the highest number of malnourished child in the world yet the number of NCDs is increasing dramatically since 1990. With 30% of all children are born underweight (17% of global total), and 48% of all under-five children are stunted, it also has the second highest prevalence of diabetes ( Danaei *et al.*, 2011) and cardiovascular disease in the world ( Basu *et al.*, 2013). India diabetic population rose to 63 million (>7% of total adult population) in 2013 which is second only to China (~92 million) and is projected to reach 100 million by 2030. Researchers suggest that demographic, epidemiological and nutrition transition is fueling the NCD epidemic in India particularly in the urban areas ( Shetty, 2002).

## The economic impacts of NCDs and nutrition transition household and national economy

NCDs are both a determinant and result of poor socio-economic status and exert an important bearing especially on the poor households as they require a long-term treatment ( Thakur *et al.*, 2011). The United Nations High Level Meeting in 2011 declared NCDs a major health, economic and development issue and one of the most significant challenges to poverty eradication in developing countries. World Economic Forum (WEF) has also identified NCDs as the second most severe threat to the global economy. According to the projections of WEF, the burden of NCDs will account for as high as $47 trillion by the year 2030 globally ( Bloom *et al.*, 2011). NCDs including diabetes are estimated to reduce GDP by up to 5% in many LDCs. Studies have shown that South Asian nations incur about a 5% loss of their annual GDP due to substandard sanitation and hygiene facilities while infections account for 20% of DALYs (Disability Adjusted Life Years) ( Basnyat & Rajapaksa, 2004). But no such study has yet been conducted focusing on the costs of NCDs. Main direct costs of NCDs include the fees for hospitalization, transportation, drugs and some indirect costs are loss of work days, absenteeism, reduced workplace productivity ( Kankeu *et al.*, 2013). Among children, obesity has a detrimental effect not only on health, but also on their psychological wellness and thus can affect lifetime earnings potential ( Roux *et al.*, 2008). In 2005, India experienced the highest loss in potentially productive years of life worldwide ( Koh-Banerjee *et al.*, 2003). Out of pocket expenditure for NCDs in India increased from 31.6% in 1995–96 to 47.3 percent in 2004, and hospital stays more than doubled during the same time ( Jaime & Lock, 2009). Among the NCDs, diabetes is the most costly and is also a major risk factor for a variety of life-threatening and expensive complications such as heart attack, renal disease, neuropathy, visual impairment ( Shariful Islam *et al.*, 2013). NCDs are also responsible for unhealthy aging and increases the burden of medical spending in later years which put huge pressure on savings and household assets. Table 4 presents a list of studies indicating the various socioeconomic and public health impacts of dietary and epidemiological transition.

**Table 4.** List of selected studies on nutrition transition in South Asia and its impacts on nutrition transition.

Study title	Reference	Study type/Scope	Conclusion
Nutrition transition in India: Secular trends in dietary intake and their relationship to diet-related NCDs.	Anoop *et al.* Journal of Diabetes 3 (2011) 278–292.	Review/Countrywide	Besides undernutrition, the epidemics of NCDs is becoming a major public health issues in both rural and urban India.
The Nutrition Transition Is Underway in India.	Paula *et al.* 2001. The Journal of Nutrition.	Original study/Countrywide	Owing to rapid urbanization and lifestyle factors associated with overweight and obesity, an increasingly large proportion of the Indian population will be at risk of overweight, obesity and associated NCDs.
The nutrition transition in India.	M Vaz *et al.* SAJCN 198 September 2005, Vol. 18, No. 2.	Review/Countrywide	India is facing a degenerative phase of the nutrition transition for which a multilevel strategies will need to be initiated at national and individual levels.
Trade Liberalization, Urbanization and Nutrition Transition in Asian Countries.	Ghose *et al.* J Nutrition Health Food Sci 1(1): 5.	Review/Countrywide	Asian countries are undergoing a rapid nutritional and epidemiological transition. Policymaking should focus on formulating strategies to minimizing social and economic implications of these diseases.
Trends in Nutrition Transition: Pakistan in Focus	Fatima *et al.* Journal of Pak Med Association. May, 2000.	Original study/Countrywide	Pakistan is in the early stages of nutrition transition with rising incidence of obesity and related NCDs. Nutrition education can play a crucial role to improve nutritional status.
Nutrition transition in Bangladesh: is the country ready for this double burden.	Shusmita *et al.* Obesity Reviews. Volume 14, pages 126–133, November 2013.	Original study/Countrywide	Nationally representative data shows that both under- and overnutrition is exiting within the same populations. Addressing overnutrition is equally if not more important than overnutrition.
Dietary Shifts in Nepal and its possible impacts on overweight and obesity.	Subedi *et al.* Proceedings of the nutrition society. January 2013.	Review/Countrywide	Nepalese dietary patterns have changed over the past forty years, especially with increased energy from plant fat, sugar and animal products coinciding with increased levels of obesity and overweight, especially in urban areas.

## Tackling the risk factors of NCDs among children

While the burden of NCDs is unquestionably going to multiply in near future, understanding of the risk factors of NCDs is still fairly poor which constitutes a major obstacle for the implementation of effective prevention strategies ( Espina *et al.*, 2013). The World Health Organization (WHO) forecasts that in next two decades there will be dramatic changes and transitions in the world's health needs, as a result of epidemiological transition ( Ahsan Karar *et al.*, 2009). A multi-sectoral policy approach must be accompanied by the adoption of national and community level nutrition and health programs to tackle this overwhelming burden of NCDs.

### Food and nutrition policy

**Figure 6.**
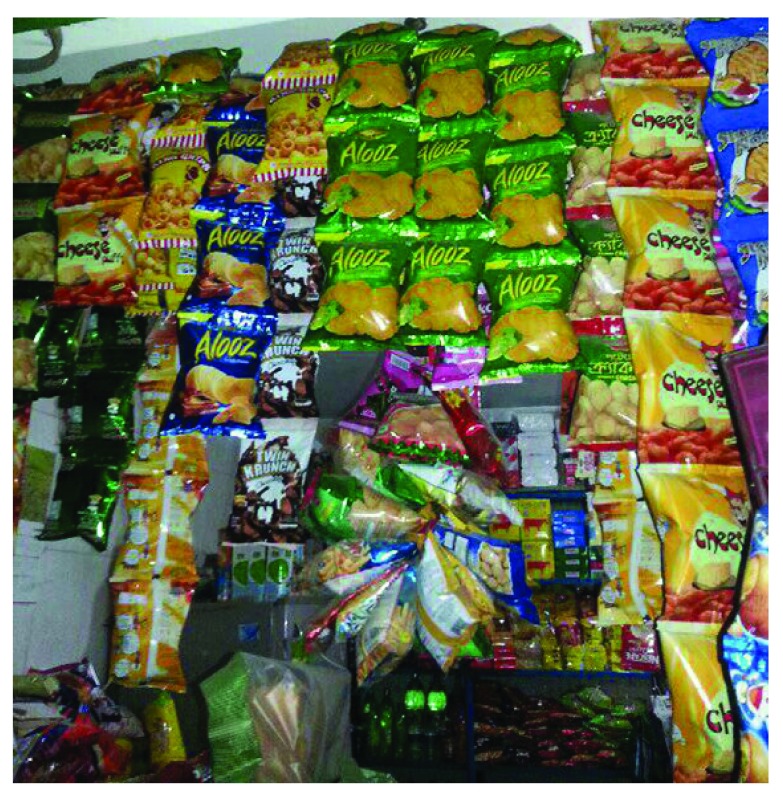
This photo was taken from a grocery shop nearby a high school in Dhaka city. The picture shows that a variety of junk foods are made available to school students. Most of these products are sold at extremely cheap prices (> $0.15). **Photo credit: Sharmistha Ghosh**

Strategic food policy making to control unhealthy dietary behaviour has been in place in many countries ( Rangel, 2013). Bolivia and Montenegro have closed down fast food shops; Hungary and Australia have imposed a surtax on soft drinks, whilst Denmark has increased tax by 25% on ice cream, chocolate and sweets products. Successful implementation of such pricing policies will require strong political will and cooperation from private sectors and civil society. As industrial control on global food systems have become the major driver of nutrition transition, more governmental regulation of food companies must aim to keep the use salt, sugar and other food additives limited according to the WHO guidelines. National food policy must also focus on increasing the access to and affordability of healthy and nutritious food and discourage the consumption of junk food. Food advertisements have a major impact on people’s dietary behaviour especially among school aged children (Barry, 2001; Vineis *et al.*, 2014). Globally, expenditure on food advertisements has almost doubled in real terms since 1980 ($512 billion in 1980 compared to $216 billion in 2004) ( Hawkes, 2006). Advertising junk foods targeted at children must be banned or reduced to a minimum. Regulating school meals is also important reducing obesity among school children. What children eat at school is hard to control but banning the sale of certain types of foods are extremely helpful. In 2005, New Jersey and California banned selling junk food and sodas in public schools, and in Ontario the government banned junk food and pop in elementary schools in 2010. In most cities in south Asia, students buy oily snacks, junk foods from school canteen or nearby shops which are both cheap and delicious. Figure 6 shows the items in a super shop near a school Dhaka city. To prevent this, school authorities must develop healthy eating programs and also ban selling junk foods in canteens and in shops.

### Promoting school-based health and nutrition programs to control childhood obesity

Schools have been promoted by policymakers, researchers and media as a strategic setting for implementing nutrition policies as it provides an inviting setting for the promotion of healthy behaviours in children which can ultimately contribute to reducing the prevalence of obesity ( Harrison & Jones, 2012). Since children spend more time in schools than in any other environment outside home, it provides an ideal workplace for developing child health intervention programs. A compelling piece of evidence suggests that physical inactivity is a strong determinant of childhood overweight and obesity which increases the risk of many chronic diseases in later life and that modest increases in physical activity have the potential to produce substantial health benefits ( Roux *et al.*, 2008). Many countries have adopted physical activity among school children. Studies have shown that apart from controlling weight, physical exercise (PE) is important because it is associated with cardiovascular benefits such as a reduction in low-density lipoproteins, prevention of hypertension, and prevention of chronic disease ( Harrison & Jones, 2012). On average, children spend around 6-8 hours per day in school. Which means they get little or no time or interest for physical activities at home after having long days. Spending a long day at school and nights with excessive home works not only make children apathetic to school but also have adverse implications for physical and mental status and social relationships. School enrollment has increased considerably in South Asia [ Table 1]. South Asia has the highest number of children in primary schools worldwide, accounting for 28% of global primary school enrollments and currently some 200 million children are enrolled in primary education. Given this huge young population, there is a risk of increasing incidence of NCDs in next few decades as obese children bear a greater risk of developing NCDs in later years. However, this risk can be turned to an opportunity for long-term prevention strategies by promoting school-based health and nutrition programs.

### Community based physical activity campaigns

People in urban areas are not only being deprived of healthy environment and healthy food, but also of adequate levels of physical activity. This is a particular concern for South Asian countries as unbalanced growth of urban areas and poor urban planning continue to aggravate the problem. Understanding the link between environment and the scope for physical activity is important to develop strategies to reduce the prevalence of obesity ( Grijalva-Eternod *et al.*, 2012; Trasande *et al.*, 2009). A large volume of literature has been published in the last decade focusing on the importance of physical activity for the effective prevention management obesity and NCDs (Lim, 2012). Most studies have highlighted that the decreasing space for physical activities is an important factor for the higher prevalence of NCDs in urban settings ( Tamosiunas *et al.*, 2014; Vaidya & Krettek, 2014). Arguably, in highly populated cities in South Asia, its hard or impossible for schools to keep sufficient space for physical activities. Besides that, children in slums and poor neighborhoods are almost always deprived of a healthy environmental space where they get little or no scope for outdoor sports and physical exercise. Though heath clubs now days are becoming popular, they are generally expensive and lack facilities for school children. Organizing frequent sports events at schools and community settings may prevent children from getting addicted to computer games. Community-based intervention programs targeting childhood obesity and nutrition-related chronic diseases must be accompanied by improving facilities for physical activities.

## Conclusions and policy recommendations

South Asian countries are undergoing various types of transitions. The study sheds some light on the impacts of nutrition transition on the emergence of NCDs in South Asia. Its highly predictable that the economic and social costs associated with nutrition transition will be felt more severely in the upcoming decades. Given the emerging epidemic of NCDs, healthcare system in South Asian countries is at a crossroads. More comprehensive studies are required to gain insight on the disease-specific social, demographic and economic determinants of NCDs in order to design more effective approaches to tackle them in the long run. In conclusion, this study proposes the adoption of following policies to address the challenges of nutrition transition and the emergence of NCDs in South Asia:

- National food policy must focus on improving peoples access to healthy and nutritious food. Better coordination between public and private sectors is required to make the policies function properly. Political will is a most crucial element to regulate and enhance food standards and make food industries abide by the regulations.
- Lack of experience and inadequate infrastructure are major hindrances to the management of NCDs in South Asia as the healthcare system is originally structured to address the acute type infectious diseases. Addressing obesity and NCDs will therefore require a restructuring of the healthcare system and for that, a well-developed NCD framework must also be put in place.
- Since NCD are going to rise, it should be incorporated in the broader development agenda to strengthen healthcare systems for NCD prevention through leveraging nationwide prevention and intervention programs, allocating sufficient resources and improving primary health care.
- School based nutrition and physical activity programs must be leveraged to reduce childhood obesity. Junk food must be banned in schools and in nearby shops and the advertising of such products must also be controlled.
- Providing information about the impacts of unhealthy diets in news papers, national TV channels and radios may help greatly to encourage healthy eating behaviour. People must be made aware of the fact that there is no silver bullets to address NCDs, and prevention remains the best cure. Health policy makers have to take measures to reduce the risky behaviours such as controlling smoking, alcoholism, use of trans-fat in restaurants and food companies, consumption of junk food.
- As the burden of obesity and NCDs is shifting towards the poor, reducing the cost of NCD drugs and ensuring equitable access to NCD care remains a high priority. Governments need to make sure that caregivers don’t profit at the cost of impoverishment of the vulnerable population. Disputes over drug patenting of life saving drugs must be dealt with by prioritizing long term health benefits over momentary economic gain.
- Building research partnerships among various sectors are essential to measure the magnitude of the problem and design a holistic approach for preventing NCDs.

## Limitations of the study

The assessment of the impact of nutrition transition is a rather complex task in the context South Asia due to highly unequal social hierarchies. The impact of recent economic growth has not been even across all societies and is criticized for accelerating income inequality. Evidently, average income is rising and health status is also improving. But the majority of the population is still living below poverty line ($1.25/day) in South Asia and it still remains home to worlds largest proportion of undernourished population. This is a highly paradoxical situation since nutrition transition is expected to have better health outcomes for these historically deprived populations who lack access to sustainable food supply and have no sight of the lifestyle changing technologies. Coexistence of over- and undernutrition is increasing in the same communities which makes it hard to develop strategies for the target population. Surtaxing certain food commodities (Meat and dairy products) might adversely affect food and nutrition security among poor households who spend a bulk of income on food. This situation calls for more comprehensive studies to make more context adjusted food and nutrition policies.
