# Supplementary material for: Nutrition transition in South Asia: the emergence of non-communicable chronic diseases
Source: F1000Res. 2015 Nov 24;4:8. Originally published 2015 Jan 12. [Version 2] doi: 10.12688/f1000research.5732.2 (PMC4706051; doi:10.12688/f1000research.5732.2)
Supplement: Peer review history

# Peer review: Referee response for version 2

**DOI:** 10.5256/f1000research.7996.r11531

**Published:** 2015-12-14

**Authors:** Saverio Stranges, Saad Siddiqui

**Affiliations:** 1 Department of Population Health, Luxembourg Institute of Health, Strassen, Luxembourg; 2 Statistics and Epidemiology Team, University of Warwick, Warwick, UK

This study is a review of the evidence concerning the nutrition transition in South Asia, and its relationship with the increasing burden of chronic illnesses.

The revised version of the manuscript is vastly improved in terms of providing extra evidence, reduction of extraneous information and the overall coherence and quality of language, and we would like to commend the author in their efforts. The comments provided by us towards the original manuscript have been addressed either through revisions in the manuscript, or via the author’s responses. Additionally, the compilation of the literature evidence in Table 4 is very welcome.

We recommend this manuscript to be indexed, since it compiles and reviews the current scientific understanding of the South Asian nutrition transition, along with addressing the various associated factors and the multifaceted policy implications.

We have read this submission. We believe that we have an appropriate level of expertise to confirm that it is of an acceptable scientific standard.

# Peer review: Referee response for version 2

**DOI:** 10.5256/f1000research.7996.r11310

**Published:** 2015-12-03

**Authors:** Ailiana Santosa

**Affiliations:** 1 Division of Epidemiology and Global Health, Umea University, Umea, Sweden

This is an interesting paper concerning nutrition transition in South Asia. 

The author has improved the manuscript, addressing comments and suggestions provided by the referees. I am quite satisfied with the revisions. Therefore, I suggest this manuscript is suitable to be accepted in this journal. I believe that the manuscript would advance and provide broad knowledge on how transition happen and its policy implication in South Asia.

I have read this submission. I believe that I have an appropriate level of expertise to confirm that it is of an acceptable scientific standard.

# Peer review: Referee response for version 1

**DOI:** 10.5256/f1000research.6127.r11199

**Published:** 2015-11-12

**Authors:** Saverio Stranges, Saad Siddiqui

**Affiliations:** 1 Department of Population Health, Luxembourg Institute of Health, Strassen, Luxembourg; 2 Statistics and Epidemiology Team, University of Warwick, Warwick, UK

**Overall Comments**

This article discusses the nutrition transition in South Asia, its influence on the nutrition profile of South Asian countries, and its relationship with the increasing burden of non-communicable diseases in the region. The author stipulates that countries in this region are mired by the dual burden of malnutrition and the new emergence of obesity and associated NCDs.

While the article and its content are poignant additions to literature on nutrition and NCDs, and the manuscript is in large part quite well-written, it could greatly benefit from a few improvements. Specifically, there are a few claims made by the author which need to be substantiated by more evidence.

**Introduction**

The section begins with a sentence that is too long, and needs to be either broken up or shortened to improve its coherence.

The author mentions several economic statistics in the first paragraph (lines 27 to 30) which need a reference. Similarly, the facts stated between lines 39 and 45 need to be substantiated with a reference.

**Methodology**

Line 2: Please add the word “review” after systematic literature.

**Demographic transition in South Asia**

Line 4: Please replace “mankind” with “global population”.

Pages 4 through to 7 present some good evidence with regards to nutrition, NCDs and socioeconomic and demographic factors.

However, under “Urbanization” on page 6, the author states that women joining the labour force “greatly influences the shift from home cooked traditional food towards precooked convenience food and fast food”. Currently, this statement is not substantiated by evidence.

**Epidemiological transition in South Asia and the burden of NCDs**

The second paragraph starts as “While in the developed countries the battle is chiefly against NCDs, people in developing countries are facing a double burden [of] malnutrition.” I believe that the first and the second halves of the sentence are not related as the word “while” would suggest. It is possible that the author intends to make a point here which is unclear at the moment, and some paraphrasing or additional information might help clarify the issue.

On the same page (7), the caption for Figure 4 ends with “with no place for fresh fruits and vegetables”. I believe that this is a non-factual interpretation of the photograph provided, since all the facts cannot be ascertained from the picture. As such, I believe that this part of the caption should be removed. Also, it would benefit the manuscript if loaded terms such as “teeming” can be removed or substituted with more neutral terms. In this specific example, the word can be removed.

In the third paragraph (p. 8, lines 13-15), the statistics on the burden of diabetes in Bangladesh need to be referenced.

**The economic impacts of NCDs**

I would like to commend the author on making a very important point about the economic burden of NCDs, especially on vulnerable households; that NCDs are both a socioeconomic determinant of health, as well as a result of poor socioeconomic status.

However, in developing countries undergoing economic transition, studies have shown that better socio-economic status puts individuals at greater risk of obesity and associated NCDs. For example:

Gouda J, Prusty R (2014) Overweight and obesity among women by economic stratum in urban India. J Health Popul Nutr 32:79–88

Pednekar MS, Hakama M, Herbert JR, Gupta PC (2008) Association of body mass index with all-cause and cause-specific mortality: findings from a prospective cohort study in Mumbai. Int J Epidemiol 37:524-535

Chhabra P, Chhabra SK (2007) Distribution and determinants of body mass index of non-smoking adults in Delhi, India. J Health Popul Nutr 25:294-301

**Tackling the risk factors of NCDs among children**

The suggestions provided at the start of the first paragraph in this section (p. 9) need to be referenced or substantiated with evidence. Have such measures been scientifically confirmed as being effective?

Statistics on the global expenditure on food advertisements needs to be referenced.

Is banning of “junk food” in schools effective as a health promotion measure for children even beyond school? The author’s suggestion is a very valid point, but it would be even better if studies which have assessed the benefits of such measures are cited.

On page 10, under “Community based physical activity campaigns”, the author mentions “most studies” as having highlighted the relationship between lack of open spaces (for physical activity) and NCDs. These studies need to be referenced here.

**Conclusions and policy recommendations**

On page 11, the final recommendation appears too presumptuous about the private sector’s responsibility in the spread of the NCD epidemic. This might be a fair point, but it can be challenged without any evidence to substantiate it. It is also too broad a claim to be included, and the recommendation to build partnerships for a holistic approach is enough on its own without the preceding sentence.

**Final comments**

I believe that the suggested improvements would advance and improve the manuscript greatly towards acceptance. I would suggest minor language edits, since there appear to be more typographical than syntactic errors, and these can be resolved through proof-reading.

We have read this submission. We believe that we have an appropriate level of expertise to confirm that it is of an acceptable scientific standard, however we have significant reservations, as outlined above.

# Response

**Published:** 2015-11-13

**Authors:** Bishwajit Ghose

**Affiliations:** Huazhong University of Science and Technology, China

Dear Professors,

Thanks indeed for your evaluation and providing valuable insights and suggestion for the article. Below is a step-by-step list of the modifications made in the revised manuscript.

**1-Introduction:**

The opening sentences was split into two.

New references were added for the suggested sentences. (39-40)

**2. Methodology:**

The word 'review' was added after 'systematic literature'.

Demographic transition in South Asia. Line 4: “Mankind” was replaced with “global population”.

Following references were added regarding demographic, socioeconomic contexts of NCDs: [41-47].

**3. Epidemiological transition in South Asia and the burden of NCDs**

The sentence was rephrased to:

*In contrast with developed countries where the disease burden is dominated by NCDs (with lower proportion of infectious diseases), countries in the developing world are facing a double burden of malnutrition*.

The following sentence was removed as suggested. Thank you!

“ The super-shops like this in the city however bear the signs of industrialized nations teeming with processed foods such as cookies, chips, and soft drinks with no place for fresh fruits and vegetables.”

New References were added (48-49).

**4. The economic impacts of NCDs**

Completely agree in this respect. Evidences on the impact of changing socioeconomic status on NCDs remain pretty much mixed and unsubstantiated. The author consulted with Prof. Barry regarding this issue and was advised that the confusion lies in the complex geographical and socio-cultural interplay which determines the variation in susceptibility to certain obesogenic factors/environments.

Authors understanding is that historically South Asian societies are extremely stratified and each stratum exhibiting varying degrees of proneness to certain diseases and risk factors. The situation is actually very perplexing and requires in-depth epidemiological studies to find an answer to this complexity.  However, explaining this issues is not in the scope of the current study and could cause deviation from the central theme.

**5. Tackling the risk factors of NCDs among children**

Governmental policy of surtaxing on tobacco has previously shown to have effectively reduced smoking rates.

1] Zhang B. The impact of tobacco tax cuts on smoking initiation among Canadian young adults. Am J Prev Med. 2006 Jun;30(6):474-9.

2] D Levy. Increasing taxes to reduce smoking prevalence and smoking attributable mortality in Taiwan: results from a tobacco policy simulation model. Tob Control. 2005 Jun; 14(Suppl 1): i45–i50.

Applying such policies on food items is hard compared to tobacco/alcohol. Few countries have only including such policies for selected food items (mentioned in the article) in their national nutrition policy to promote healthy eating habits. Their effectiveness remains to be studied.

In the context of South Asia such policies may sound paradoxical since the rate of poverty and undernutrition is still very high. However, given the rising burden of morbidity and mortality from NCDs, such policies deserve due attention in the public health agenda. In theory, raising prices should reduce the consumption of a given product. However, whether or not it will translate to lower prevalence of NCDs in the long term will also require long-term trials. Authors proposed these policies based on the synthesis of policy literature from developing countries, not of epidemiological findings.

Reference 51 was added for the comment as suggested.

Statistics on the global expenditure on food advertisements was given reference no [50].

Majority of the studies have actually focused on the impact of selling of junk food inside schools and have suggested to ban selling junk foods both on campus and in shops nearby.  However, evidences from studies focusing exclusively on shops away from school are not yet available.

Page 10: References 52-53 were added.

**Conclusions**

The sentence was removed. Thank you!

The manuscript was revised for typographical error was corrected where applicable. Thank you!

# Peer review: Referee response for version 1

**DOI:** 10.5256/f1000research.6127.r10887

**Published:** 2015-11-02

**Authors:** Ailiana Santosa

**Affiliations:** 1 Division of Epidemiology and Global Health, Umea University, Umea, Sweden

This is a widespread, well-balanced and comprehensive review of study over the last two decades on the nutrition transition. The manuscript reads well and has important findings. The introduction clearly defines the purpose of the manuscript and the relevance to the policy practice.

However, the author does not clearly describe the literature search strategies in the methods section as he mentioned it in the objective of the study. He stated that he used several electronic databases including COCHRANE library. Has he fulfilled the COCHRANE checklist? He also did not clearly state the inclusion and exclusion criteria. Even he stated that there were no special exclusion criteria (why there are no special exclusion criteria?). My concerns are while the method of review paper is unclear, it will dilute the findings and the discussion later. I found that some of findings are diluted and unclear.

In revising the manuscript, the author should:

1. Divide the published studies between review studies and empirical studies, and bring to light emerging evidence from existing review studies and clarify what new evidence is learnt from their review.
2. Divide evidence regarding different terms of transition that he focused on and explanatory factors contributing to the nutrition transition in South Asia.
3. In the "Methods" section, clarify which are the inclusion and exclusion criteria, the agreement for selecting the published articles used for review, the indicator(s) are used to evaluate the nutrition transition or lack of it, and attempt in the presentation of the findings to interpret the sensitivity of the findings to alternative specification of indicator for measuring the timing and stage of transitions.
4. Summarise in a table the evidences (and number of studies) for the nutrition transition by country coverage (rural, urban, sub-national, national); then address the extent to which the predictive article expected from the nutrition transition has varied across study countries.

I have read this submission. I believe that I have an appropriate level of expertise to confirm that it is of an acceptable scientific standard, however I have significant reservations, as outlined above.

# Response

**Published:** 2015-11-13

**Authors:** Bishwajit Ghose

**Affiliations:** Huazhong University of Science and Technology, China

Dear Professor,

I wish to extend my sincere thanks for your valuable comments and suggestions regarding the paper.

The contribution of the present review is that it reaffirms the findings of past studies by cross verifying from multiple studies conducted on the individual countries and proposes a set of policy instruments.

As shown in the tables and figures, the findings on the drivers and outcomes of dietary transition were consistent for the countries included in the study. 

Cochrane Library was searched for this study, but the database provided no directly relevant article itself. Reference lists however were searched manually for articles of relevance and were sourced from the publisher.  

Authors mentioned that there is no such study in the context of South Asia as yet, and  the literature resource is notably meagre. Which is why no special exclusion criteria was applied. Broadening the inclusion criteria facilitated the triangulation and cross-verification of evidences from different sources. Almost of the articles were reviews and no clinical trials were available on the topic.

With regard to comment 2, the review summarizes the proximal sociodemographic and economic factors which are driving the dietary and epidemiological transition and have presented concrete evidences on the shifting epidemiological pattern in South Asia. It recognizes the absence of nationally representative studies on exploring more subtle and context specific underlying causes of dietary transition. Knowledge from more in-depth studies is crucial and could be leveraged to develop national nutrition policies targeting the rising burden of NCDs. Previous studies were limited in their scope of showing that nutrition transition is underway in these countries, however missed the opportunity to propose policies based on their findings. To this end, apart from summarizing the past studies, the main aim of this review was to the synthesize a range of policy measures based on studies and policy papers in countries who have long experienced nutrition transition and NCDs prevention policies.

The methodology section was rewritten as suggested. Thank you!

With regard to part of comment 1 and 4, a new table (4) was included providing the information as suggested.  Thank you!
